# Supplementary material for: DMRforPairs: identifying Differentially Methylated Regions between unique samples using array based methylation profiles
Source: BMC Bioinformatics. 2014 May 15;15:141. doi: 10.1186/1471-2105-15-141 (PMC4046028; doi:10.1186/1471-2105-15-141)
Supplement: Additional file 2 — DMRforPairs output for the comparison of A431-MCF7 and NA17018-NA17105. Please start from the HTML files in each folder. Available via the BMC Bioinformatics website. [file 1471-2105-15-141-S2.zip › 1394847754114233_MOESM2_ESM/A431_MCF7/figures/19231.pdf]

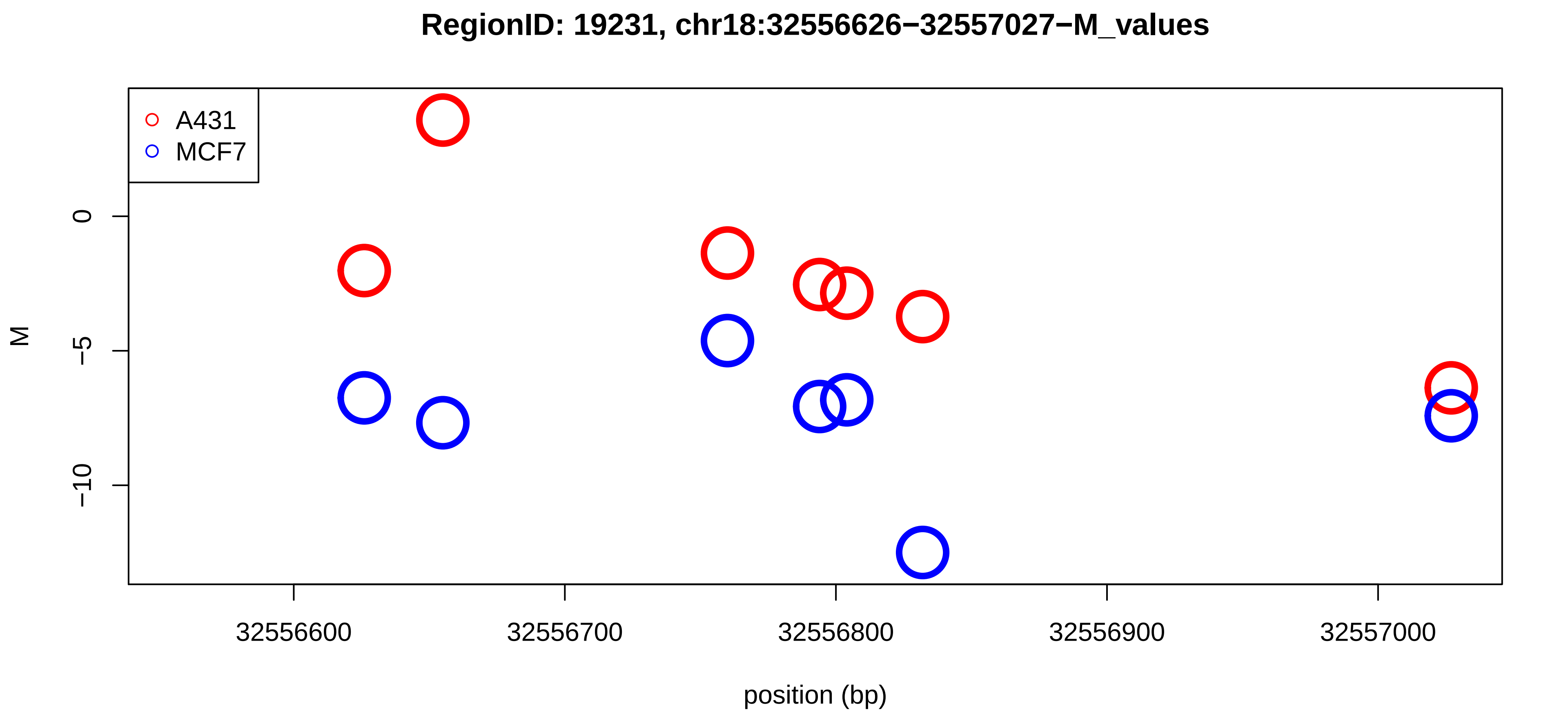

RegionID: 19231, chr18:32556626−32557027−M_values
A431
MCF7
0
−5
−10
M
32556600
32556700
32556800
32556900
32557000
position (bp)

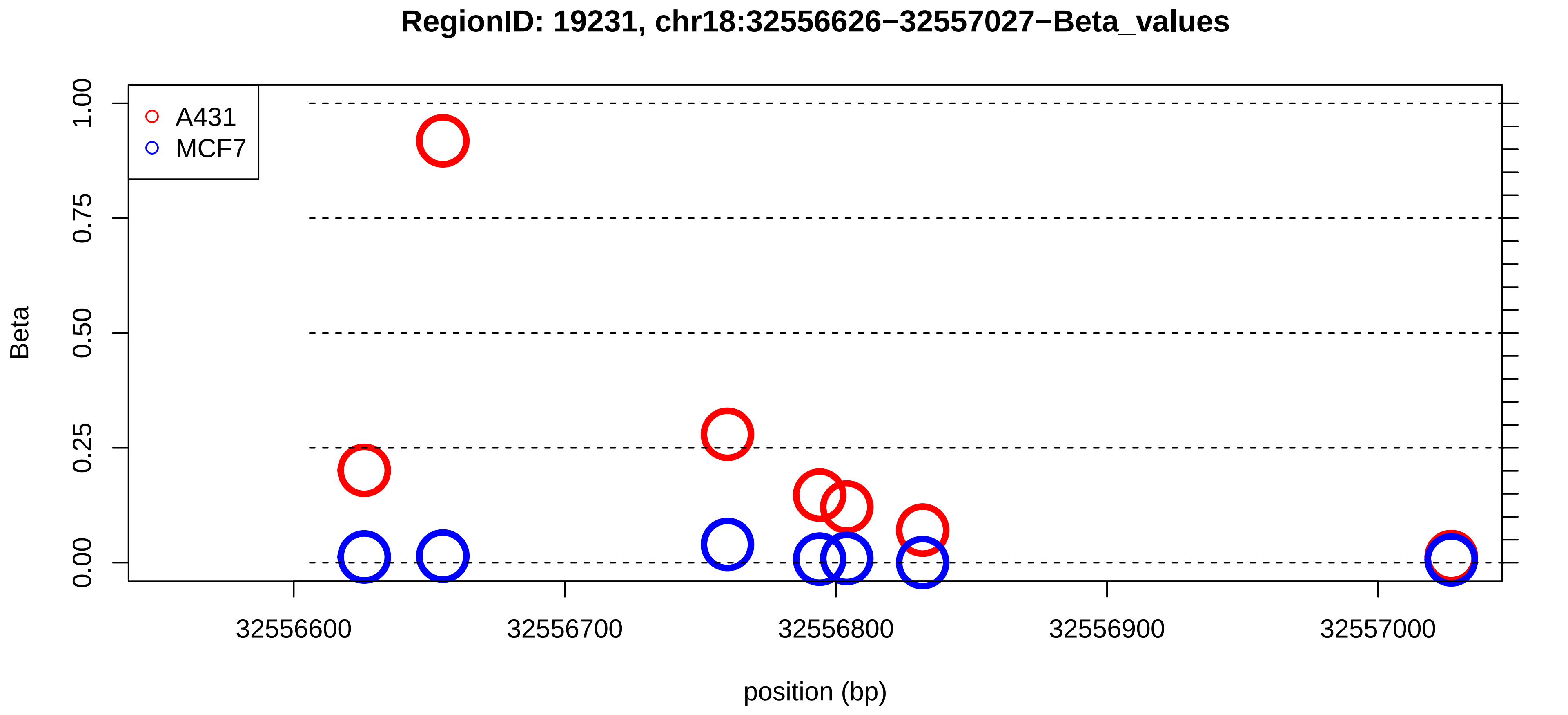

RegionID: 19231, chr18:32556626−32557027−Beta_values
A431
MCF7
1.00
0.75
0.50
0.25
0.00
Beta
32556600
32556700
32556800
32556900
32557000
position (bp)

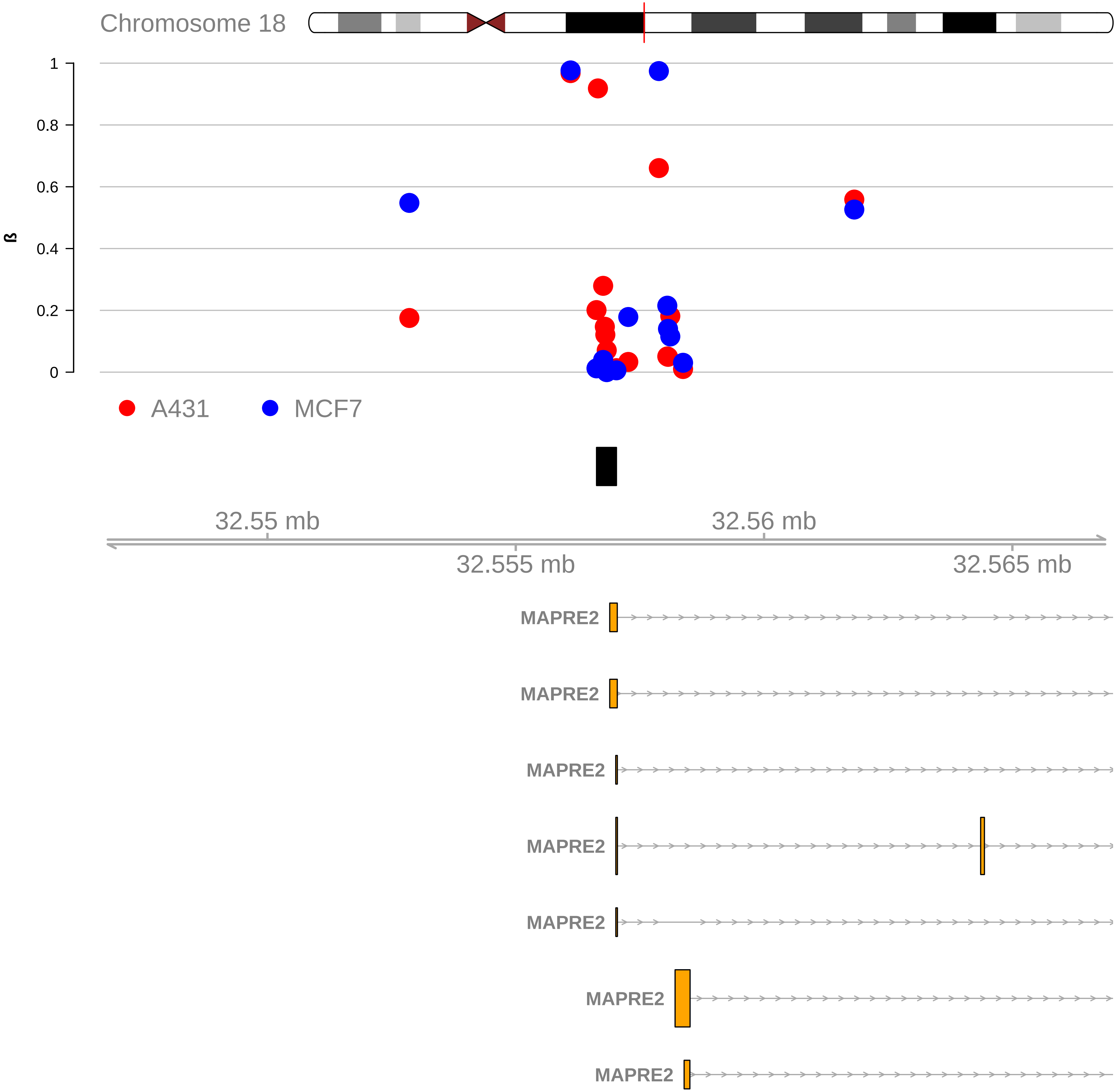

Chromosome 18
1
0.8
0.6
0.4
0.2
0
ß
A431
MCF7
32.55 mb
32.56 mb
32.555 mb
32.565 mb
MAPRE2
MAPRE2
MAPRE2
MAPRE2
MAPRE2
MAPRE2
MAPRE2
